# Age, Pulse, Urea, and Albumin Score: A Tool for Predicting the Short-Term and Long-Term Outcomes of Community-Acquired Pneumonia Patients With Diabetes

**DOI:** 10.3389/fendo.2022.882977

**Published:** 2022-06-01

**Authors:** Chun-Ming Ma, Ning Wang, Quan-Wei Su, Ying Yan, Si-Qiong Wang, Cui-Hua Ma, Xiao-Li Liu, Shao-Chen Dong, Na Lu, Li-Yong Yin, Fu-Zai Yin

**Affiliations:** ^1^ Department of Endocrinology, The First Hospital of Qinhuangdao, Qinhuangdao, China; ^2^ Department of Internal Medicine, Hebei Medical University, Shijiazhuang, China; ^3^ Department of Internal Medicine, Chengde Medical College, Chengde, China; ^4^ Department of Internal Medicine, Hebei North University, Zhangjiakou, China; ^5^ Clinical Laboratory, The First Hospital of Qinhuangdao, Qinhuangdao, China; ^6^ Respiratory and Critical Care Medicine, The First Hospital of Qinhuangdao, Qinhuangdao, China; ^7^ Department of Neurology, The First Hospital of Qinhuangdao, Qinhuangdao, China

**Keywords:** community-acquired pneumonia, APUA score, short-term outcome, long-term outcome, diabetes

## Abstract

**Objective:**

The predictive performances of CURB-65 and pneumonia severity index (PSI) were poor in patients with diabetes. This study aimed to develop a tool for predicting the short-term and long-term outcomes of CAP in patients with diabetes.

**Methods:**

A retrospective study was conducted on 531 CAP patients with type 2 diabetes. The short-term outcome was in-hospital mortality. The long-term outcome was 24-month all-cause death. The APUA score was calculated according to the levels of Age (0-2 points), Pulse (0-2 points), Urea (0-2 points), and Albumin (0-4 points). The area under curves (AUCs) were used to evaluate the abilities of the APUA score for predicting short-term outcomes. Cox regression models were used for modeling relationships between the APUA score and 24-month mortality.

**Results:**

The AUC of the APUA score for predicting in-hospital mortality was 0.807 in patients with type 2 diabetes (*P*<0.001). The AUC of the APUA score was higher than the AUCs of CURB-65 and PSI class (*P*<0.05). The long-term mortality increased with the risk stratification of the APUA score (low-risk group (0-1 points) 11.5%, intermediate risk group (2-4 points) 16.9%, high risk group (≥5 points) 28.8%, *P*<0.05). Compared with patients in the low-risk group, patients in the high-risk group had significantly increased risk of long-term death, HR (95%CI) was 2.093 (1.041~4.208, *P*=0.038).

**Conclusion:**

The APUA score is a simple and accurate tool for predicting short-term and long-term outcomes of CAP patients with diabetes.

## Background

Community-acquired pneumonia (CAP) is a common respiratory disease. The outcomes vary enormously between different CAP patients, from rapid recovery to death. CURB-65 and the pneumonia severity index (PSI) are two classical tools for evaluating the severity of CAP, and are recommended by several CAP guidelines (1, 2). A previous meta-analysis showed that the area under the curves (AUCs) of CURB-65 and PSI were about 0.8 for predicting 30-day mortality in hospitalized CAP patients (3).

Diabetes is another global problem. About 463 million adults were living with diabetes worldwide in 2019. Patients with diabetes have an increased risk of up to 1.4 for CAP (4). Patients with CAP who have diabetes have higher in-hospital and long-term mortality (5, 6). However, the predictive performances of CURB-65 and PSI were poor in patients with diabetes. The AUCs of CURB-65 and PSI were only 0.655~0.727 in patients with diabetes (7, 8).

Recently, we developed the APUA model for predicting mortality of CAP, adapted for patients with type 2 diabetes mellitus (T2DM). The APUA model is based on serum albumin, and consists of age, pulse, urea, and albumin. The predictive performance of the APUA model was better than the CURB-65 and PSI class in CAP patients with T2DM. However, this study had some limitations. First, we only performed an internal validation in this study. Second, the relationship between the APUA model and long-term outcome of CAP was unknown. Third, the calculation of the APUA model needed a nomogram or web calculator (8).

Therefore, in this study, the APUA model was simplified as the APUA score and the performance of the APUA score for predicting short-term and long-term outcomes of CAP was proved in patients with diabetes.

## Methods

### Subjects

The patients in this study were from a survey on in-hospital mortality of CAP in patients with T2DM at our hospital. The details of the study were described in a published paper (8). All subjects were adult inpatients with T2DM, who were hospitalized due to CAP between January 2015 and December 2018. Patients with type 1 diabetes, other specific types of diabetes, no clear type classification, pre-diabetes, pregnancy, and obstetric infection were excluded. Patients with missing clinical data on serum albumin, CURB-65, and PSI were also excluded. There were 531 T2DM patients with CAP (305 men and 226 women), aged 71.7 ± 11.7 years, enrolled. This study was approved by the ethics committee of the First Hospital of Qinhuangdao (the ethical approval number is 2021A006).

### Data Collection

Initial data after admission were extracted from the Hospital Information System. Sociodemographic variables included age, sex, and ethnicity. Clinical data included the diagnosis and classification of diabetes, neoplastic disease, liver disease, congestive heart failure, cerebrovascular disease, and renal disease. Data on chronic obstructive pulmonary disease (COPD) and asthma and coronary heart disease (CHD) were also collected. Physical examination included mental status, respiratory rate, blood pressure, temperature, and pulse. Laboratory data included arterial pH, PaO_2_, SaO_2_, urea, albumin, sodium, glucose, and hematocrit. Pleural effusion was also collected.

The scores of CURB-65 [confusion, urea >7 mmol/L, respiratory rate ≥30/min, blood pressure (systolic blood pressure <90 mmHg or diastolic blood pressure ≤60 mmHg) and age ≥65years] and PSI were calculated. According to the PSI score, patients were classified into five risk classes (9, 10).

The APUA model was simplified as the APUA score. The simplified method of the APUA model is shown in **Table 1**. The APUA score includes Age (0-2 points), Pulse (0-2 points), Urea (0-2 points), and Albumin (0-4 points). The total points were 10 points. We respect the conservative convention of at least 10 outcome events per predictor variable (11). The APUA score included four predictor variables. Therefore, at least 40 outcome events were needed.

**Table 1:** The simplified method of the APUA model.

Variables		APUA model	APUA score
Age (years)	<65	0	0
	65-84	31.68277	1
	≥85	62.01556	2
Pulse (/min)	<125	0	0
	≥125	52.73172	2
Urea (mmol/L)	<11	0	0
	≥11	49.77131	2
Albumin (g/L)	≥35	0	0
	25-34.9	63.22964	2
	<25	100	4
Total points			10

### Outcome

The short-term outcome was in-hospital mortality. Mechanical ventilation, ICU admissions, septic shock, and respiratory failure were also analyzed.

The long-term outcome was 24-month all-cause death. Patients who died during hospitalization were excluded. The 24-month data were retrieved from the records of the return visits to patients. The need for informed consent was waived because of the retrospective, medical record-based design of the study.

### External Validation

In the current study, we obtained data from the National Population Health Data Center (NPHDC) (https://www.ncmi.cn//phda/dataDetails.do?id=CSTR:A0006.11.Z02B6.202012.119.V1.0). The raw data were shared by Lili Zhao. The detail of the study was described in a published paper. A prospective, multi-center study was conducted between January 2017 and December 2018 in China (12). There were 366 CAP patients entered in this study. Among these patients, 107 diabetes patients were included in our study. The primary outcome was 30-day mortality. We downloaded the raw data for secondary analysis.

### Statistical Analyses

All analyses were performed using the SPSS 24.0 statistical software (SPSS 24.0 for Windows; SPSS, Inc., Chicago, IL). Numerical variables were reported as mean ± standard deviation. Comparisons were conducted between groups using variance analysis. CURB-65 and PSI were not normally distributed, they were expressed as medians (interquartile range). Comparisons were conducted between groups using the Kruskal-Wallis test. Categorical data were reported as abnormal subjects (%) and chi-square tests were used. The AUCs for CURB-65, PSI, and APUA score were drawn by receiver operating characteristic curve (ROC curve) analysis. AUCs were used to evaluate the abilities of CURB-65, PSI, and APUA score for predicting short-term outcome. The ROC curve analysis was performed with MedCalc15.2.2 software (Ostend, Belgium). Cox regression models were used for modeling relationships between the APUA score and 24-month mortality. *P*<0.05 was considered statistically significant.

## Results

**Figure 1:**
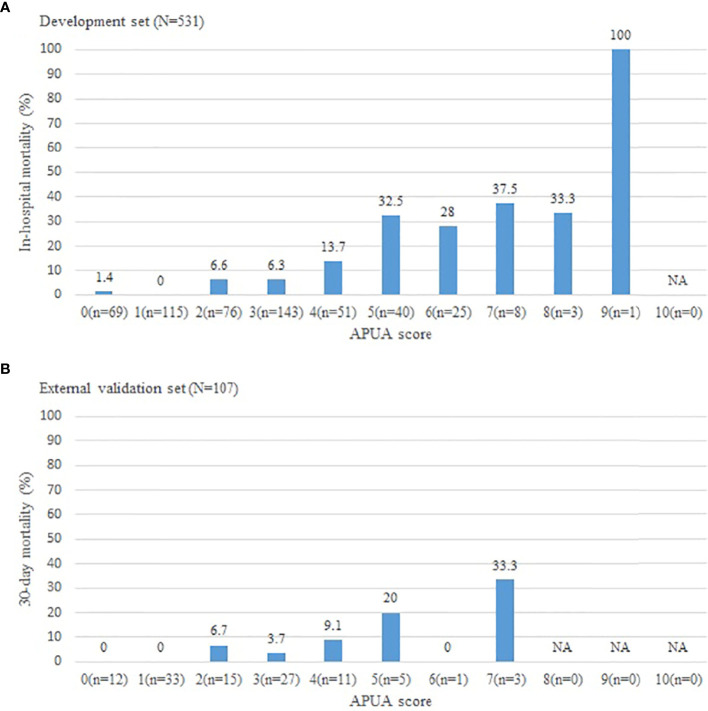
The short-term outcome in community acquired pneumonia patients with different APUA score. **(A)**: Development set; **(B)**: External validation set. APUA score: Age, Pulse, Urea, and Albumin (APUA) score. NA, Not Applicable.

In these CAP patients with T2DM, 47 patients (8.9%) died. In-hospital mortality increased with the APUA score in CAP patients with T2DM (*P*<0.001) (**Figure 1A**). According to APUA score, patients were classified into three risk stratifications. Patients with APUA score 0-1 point was defined as the low-risk group, 2-4 points was defined as the intermediate risk group, and ≥5 points was defined as the high-risk group. Septic shock, respiratory failure, mechanical ventilation, ICU admissions, and in-hospital mortality increased with the risk stratification defined as the APUA score (*P*<0.001) (**Table 2**).

**Table 2:** Characteristics of community acquired pneumonia patients with different APUA score.

Variables	Low-risk group (APUA 0-1 poitnt)	Intermediate risk group (APUA 2-4 poitnts)	High-risk group (APUA ≥5 poitnts)	*F* or *χ* ^2^	*P*
Development set (N=531)					
Sex (men/women)	94/90	165/105	46/31	3.089	0.079
Age (years)	68.0 ± 10.2	72.7 ± 12.0	77.2 ± 10.9	20.340	<0.001
CURB-65	1.0 (0.0~2.0)	1.0 (1.0~2.0)	2.0 (2.0~3.0)	118.456	<0.001
PSI	77.0 (64.0~89.8)	98.0 (82.0~116.0)	133.0 (115.5~149.0)	177.265	<0.001
Septic shock [n (%)]	1 (0.5)	16 (5.9)	19 (24.7)	42.067	<0.001
Respiratory failure [n (%)]	20 (10.9)	69 (25.6)	34 (44.2)	35.233	<0.001
Mechanical ventilation [n (%)]	6 (3.3)	36 (13.3)	27 (35.1)	44.919	<0.001
ICU admissions [n (%)]	5 (2.7)	48 (17.8)	39 (50.6)	80.331	<0.001
In-hospital mortality [n (%)]	1 (0.5)	21 (7.8)	25 (32.5)	57.885	<0.001
External validation set (N=107)					
Sex (men/women)	28/17	38/15	5/4	0.084	0.773
Age (years)	69.0 ± 10.8	75.4 ± 14.4	72.3 ± 7.6	3.221	0.044
CURB-65	1.0 (1.0~1.0)	1.0 (1.0~2.0)	2.0 (1.5~3.0)	21.852	<0.001
PSI	75.0 (66.5~83.5)	90.0 (83.5~112.0)	111.0 (101.0~140.5)	30.287	<0.001
Septicopyemia [n (%)]	2 (4.4)	5 (9.4)	4 (44.4)	8.334	0.004
ARDS [n (%)]	0 (0.0)	2 (3.8)	1 (11.1)	3.504	0.061
Severe CAP [n (%)]	3 (6.7)	11 (20.8)	6 (66.7)	14.729	<0.001
30-day mortality [n (%)]	0 (0.0)	3 (5.7)	2 (22.2)	7.199	0.007

CURB-65 and PSI were not normally distributed, they were expressed as medians (IQR). Comparisons were conducted between groups using the Kruskal-Wallis test. PSI, pneumonia severity index; ICU, intensive care unit; ARDS, acute respiratory distress syndrome; CAP, community acquired pneumonia; IQR, interquartile range.

The AUC of the APUA score for predicting in-hospital mortality was 0.807 in patients with T2DM (*P*<0.001). The AUC of the APUA score was higher than the AUCs of CURB-65 and PSI class (*P*<0.05) (**Table 3**). When the cutoff point of the APUA score was defined as ≥ 2 points, the sensitivity was 97.9% and the specificity was 37.8% (**Table 4**).

**Table 3:** The accuracy of APUA, CURB-65, and PSI score for evaluating the risk of short-term outcome in community acquired pneumonia patients with diabetes.

Variables	AUC (95%CI)	SE	*P*	*P* ^*^
Development set (N=531)				
APUA	0.807 (0.771~0.840)	0.031	<0.001	
CURB-65	0.677 (0.636~0.717)	0.040	<0.001	<0.001
PSI	0.716 (0.675~0.754)	0.035	<0.001	0.012
External validation set (N=107)				
APUA	0.808 (0.720~0.878)	0.092	<0.001	
CURB-65	0.721 (0.626~0.803)	0.114	0.053	0.601
PSI	0.769 (0.677~0.845)	0.088	0.002	0.751

Development set: in-hospital mortality; external validation set: 30-day mortality. AUC, area under curve; CI, confidence interval; SE, standard error; PSI, pneumonia severity index. * Compared with APUA.

**Table 4:** The sensitivities, specificities, and Youden’s index of the AUPA score for evaluating the short-term outcome in community acquired pneumonia patients with diabetes.

APUA Score	Development set (N=531)			External validation set (N=107)		
	Sen (%)	Spe (%)	Youden’s index	Sen (%)	Spe (%)	Youden’s index
0	100	0	0	100	0	0
1	97.9	14.1	0.119	100	11.8	0.118
2	97.9	37.8	0.357	100	44.1	0.441
3	87.2	52.5	0.397	80	57.8	0.378
4	68.1	80.2	0.483	60	83.3	0.433
5	53.2	89.3	0.425	40	93.1	0.331
6	25.5	94.8	0.204	20	97.1	0.171
7	10.6	98.6	0.092	20	98.0	0.180
8	4.3	99.6	0.039	0	100	0
9	2.1	100	0.021	NA	NA	NA
10	0	100	0	NA	NA	NA

Sen, sensitivity; Spe, specificity.

NA, Not Applicable.

The results were similar in an external validation set. The 30-day mortality increased with the risk stratification defined as the APUA score (*P*<0.01) (**Figure 1B**) (**Table 2**). The AUC of the APUA score for predicting 30-day mortality was 0.808 in patients with diabetes (*P*<0.001) (**Table 3**). When the cutoff point of the APUA score defined as ≥ 2 points, the sensitivity was 100.0% and the specificity was 44.1% in patients with diabetes (**Table 4**).

**Figure 2:**
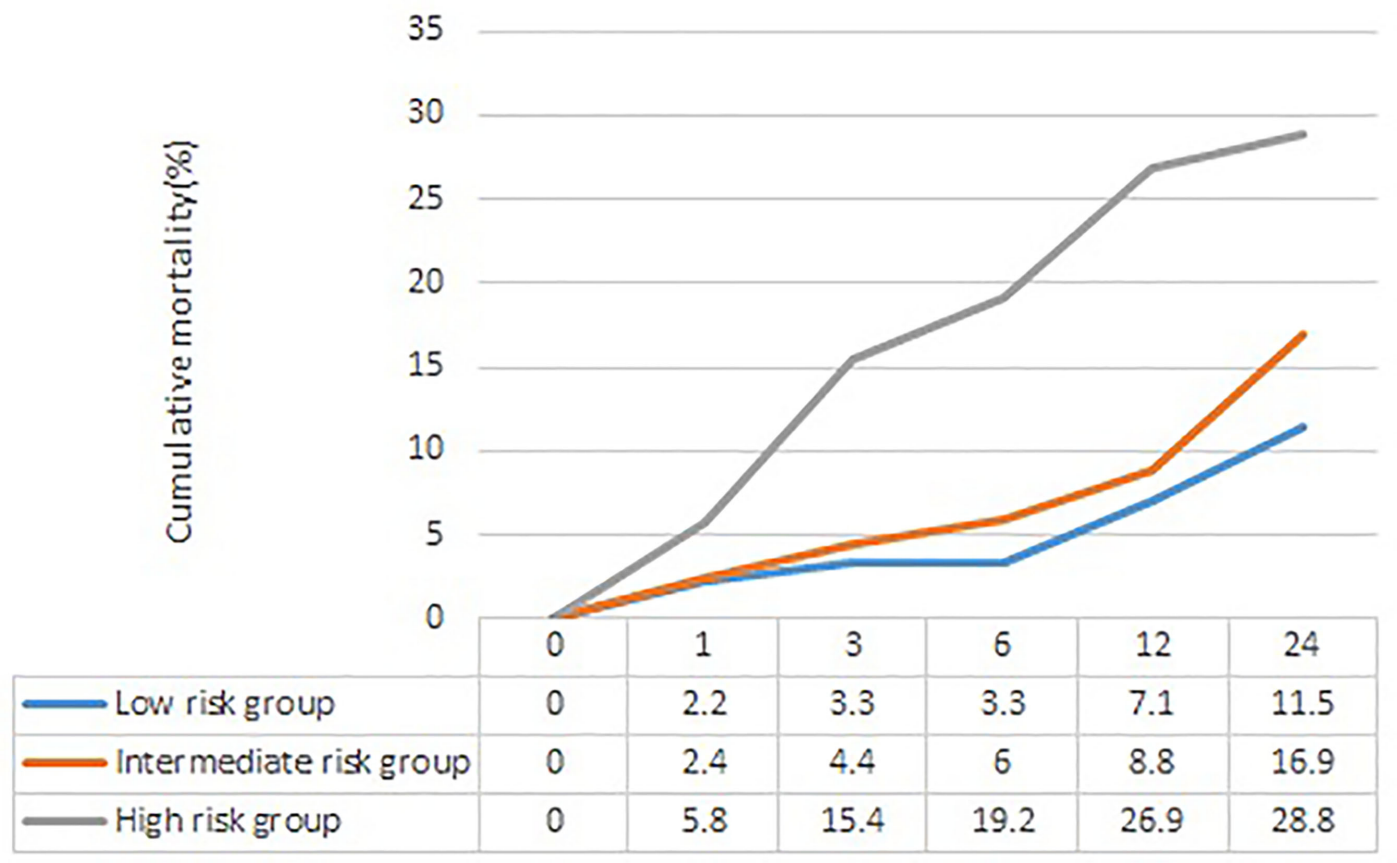
The cumulative mortality of different risk stratification of APUA score in community acquired pneumonia patients with type 2 diabetes. APUA score: **A**ge, **P**ulse, **U**rea, and **A**lbumin (APUA) score.

The section about long-term outcome enrolled 484 CAP inpatients with T2DM survival to hospital discharge (278 men and 206 women), age 71.2 ± 11.6 years. In these patients, 78 (16.1%) died at the end of the 24-month follow-up. The long-term mortality increased with the risk stratification of the APUA score (low-risk group 11.5%, intermediate risk group 16.9%, high-risk group 28.8%, *P*<0.05) (**Figure 2**) (**Table 5**). Compared with patients in the low-risk group, patients in the high-risk group had significantly increased risk of long-term death, HR (95%CI) was 2.093 (1.041~4.208, *P*=0.038) after adjusting for sex, neoplastic disease, chronic obstructive pulmonary disease, asthma, coronary heart disease, heart failure, renal disease, and cerebrovascular disease (**Table 5**).

**Table 5:** Cox proportional hazards regression analysis of risk stratification of APUA score and long-term outcome in community acquired pneumonia patients with type 2 diabetes.

APUA score	24-month mortality [n(%)]	Model 1		Model 2	
		HR (95%CI)	*P*	HR (95%CI)	*P*
Low-risk group (N=183)	21 (11.5)	1		1	
Intermediate risk group (N=249)	42 (16.9)	1.494 (0.885~2.523)	0.133	1.354 (0.792~2.312)	0.268
High-risk group (N=52)	15 (28.8)	2.923 (1.507~5.673)	0.002	2.093 (1.041~4.208)	0.038

Model 1: Univariate analysis, Model 2: Multivariate analysis, adjusted for sex, neoplastic disease, chronic obstructive pulmonary disease, asthma, coronary heart disease, heart failure, renal disease, and cerebrovascular disease as independent variables. HR, hazard ratio; CI, confidence interval.

## Discussion

In this study, we developed the APUA score based on serum albumin. Hypoalbuminemia increased the risk of hospitalization for CAP (13). Decreased albumin levels were also associated with prolonged time to reach clinical stability, ICU admission, the need for mechanical ventilation, and 30-day mortality (14). We found that the severity of CAP increased with the APUA score. In patients with APUA ≥5 points, one in three patients needed mechanical ventilation, and half of the patients stayed in ICU and nearly one in three patients died in-hospital.

In our previous study, we developed the APUA model for predicting mortality of CAP, adapted for patients with T2DM (8). In this study, the APUA model was simplified as the APUA score. In T2DM patients with CAP, the APUA score was superior to CURB-65 and PSI class. In an external validation set, the APUA score was similar to CURB-65 and PSI score in the general population and had the maximal AUC in diabetic patients. However, PSI class is a complicated scoring system. The APUA score is relatively simpler and consists of only four risk factors. This means that the simple APUA score can be used in CAP patients with diabetes.

CAP affected the long-term outcome after initial recovery. In 1993, Brancati reported that 32% CAP patients died over the next 24 months (15). Subsequently, clinicians pay more attention to the long-term outcome of CAP inpatients after discharge. In a CAP-China cohort, the long-term mortality is higher among patients with CAP compared with the age-adjusted general population (16). Diabetes also increased the mortality of CAP after several years (6).

In this study, long-term mortality of CAP increased with the risk stratification defined as the APUA score in patients with T2DM. In T2DM patients with APUA score ≥5 points survival to hospital discharge, nearly 30% patients died during the 24-month follow-up period. Tokgoz Akyil et al. found that blood urea nitrogen/albumin ratio was significantly associated with long-term mortality of CAP (17). A 5-year prospective follow-up study from Norway found that aging and low serum albumin level at admission were independent risk factors of all-cause mortality (18). The APUA score is based on serum albumin, and consists of age, pulse, urea, and albumin. The results of our study confirmed that the APUA score can be used for predicting the long-term outcome of CAP in patients with T2DM.

In patients with CAP, the short-term outcome is mainly dependent on pneumonia severity; however, long-term outcome is considered to depend on comorbidity (18, 19). Many comorbidities, such as cardiovascular disease, cerebrovascular disease, neoplasms, and COPD correlated with increased mortality in CAP patients (20). To remove these potential confounders, multifactor analysis was used. After adjustment for potential confounders, we found that the APUA score was independently associated with long-term mortality of CAP in patients with T2DM.

Compared with the APUA model, the APUA score has several advantages. First, the APUA score is more easily calculated. The APUA score does not need a nomogram or web calculator. Second, the accuracy of the APUA score was proved in an external validation set. Third, the relationship between APUA score and long-term outcome was validated in the cohort study.

This study had some limitations. First, the pathogens were not analyzed in our study. Second, the external validation set did not include pulse. Heart rate was a substitute for pulse in the external validation set. Third, the relationship among APUA score, short-term outcomes, and long-term outcomes should be validated in other populations.

## Conclusion

In summary, the APUA score is a simple and accurate tool for predicting short-term and long-term outcomes of CAP patients with diabetes.

## Data Availability Statement

The raw data supporting the conclusions of this article will be made available by the authors, without undue reservation.

## Ethics Statement 

The need for informed consent was waived because of the retrospective, medical record-based design of the study.

## Author Contributions

Study design: F-ZY. Writing: C-MM. Data analysis: NW. Data collection: Q-WS, YY, S-QW, C-HM, X-LL, S-CD, NL, and L-YY. All authors contributed to the article and approved the submitted version.

## Funding

This work was supported by the Hebei Province Key Research Projects of (21377749D).

## Conflict of Interest

The authors declare that the research was conducted in the absence of any commercial or financial relationships that could be construed as a potential conflict of interest.

## Publisher’s Note

All claims expressed in this article are solely those of the authors and do not necessarily represent those of their affiliated organizations, or those of the publisher, the editors and the reviewers. Any product that may be evaluated in this article, or claim that may be made by its manufacturer, is not guaranteed or endorsed by the publisher.
